# Carbonate Acidizing: The Effect of Corrosion Inhibitor and Emulsion Preventer on Pore Volume to Breakthrough

**DOI:** 10.1021/acsomega.5c12206

**Published:** 2026-03-11

**Authors:** Guilherme Mentges Arruda, Ernani Dias da Silva Filho, Myllena Rosana de Araújo Medeiros, Alicia Luzia da Costa Silva, Leonardo José do Nascimento Guimarães, José Antonio Barbosa, Mateus Palharini Schwalbert, Marcos Allyson Felipe Rodrigues

**Affiliations:** † Department of Chemical Engineering, 28123 Federal University of Rio Grande do Norte, 59072-970 Natal, RN, Brazil; ‡ Department of Petroleum Engineering, Federal University of Rio Grande do Norte, 59064-970 Natal, RN, Brazil; § Institute of Chemistry, Federal University of Rio Grande do Norte, 59078-970 Natal, RN, Brazil; ∥ Civil Engineering Post-Graduation Program, 28116 Federal University of Pernambuco, Recife 50740-530, PE, Brazil; ⊥ Department of Geology, Federal University of Pernambuco, 50740-530 Recife, PE, Brazil; # 42506 Petróleo Brasileiro S.A – PETROBRAS, 21941-915 Rio de Janeiro, RJ, Brazil

## Abstract

Acid stimulation of carbonate rocks aims to create channels within the rock, known as wormholes, to restore or enhance permeability, increasing production. Among the acids used in this type of treatment, 15 wt % hydrochloric acid (HCl) stands out due to its high reactivity, formation of soluble reaction products in aqueous media, and cost-effectiveness. To minimize corrosion of metallic structures and avoid emulsion formation after contact with formation oil, acid solutions are commonly prepared with additives such as corrosion inhibitors and emulsion preventers. To evaluate the influence of these additives on wormhole formation, this study performed core flooding experiments at different injection rates to construct pore volume to breakthrough (PV_bt_) curves, both in the presence and absence of these additives. The tests employed 15 wt % HCl solutions, with and without additives, using Indiana Limestone rocks containing 98.57% calcite. The experiments were conducted in a core flooding system under an injection rate range of 0.25–16 mL/min, at 25 °C, with a confining pressure of 2000 psi and back pressure of 1200 psi. After the tests, the cores were analyzed by X-ray microcomputed tomography to evaluate wormhole formation. The results indicated that additives reduced PV_bt_ values at low flow rates, suggesting slower reaction kinetics and higher wormhole formation efficiency. The presence of additives decreased the optimal interstitial velocity by approximately 77% (from 0.91 to 0.21 cm/min), indicating that they provide better reaction control and enhance treatment efficiency. Moreover, micro-CT images confirmed the formation of dominant wormholes at almost all flow rates in the presence of additives, whereas in their absence (e.g., at 0.5 mL/min), the sample collapsed before breakthrough. The Buijse–Glasbergen model provided a good fit to the experimental data (*R* ^2^ = 0.99) for the additive-free curve. For the additive-containing system, however, an empirical adjustment of the model exponent was required to improve the correlation (from *R* ^2^ = 0.85 to *R* ^2^ = 0.93). The results demonstrate that these additives not only inhibit corrosion and prevent emulsions but also controlled the reactivity effect. This behavior significantly broadens the scope of their application, reinforcing their strategic importance in carbonate reservoir treatments.

## Graphical abstract

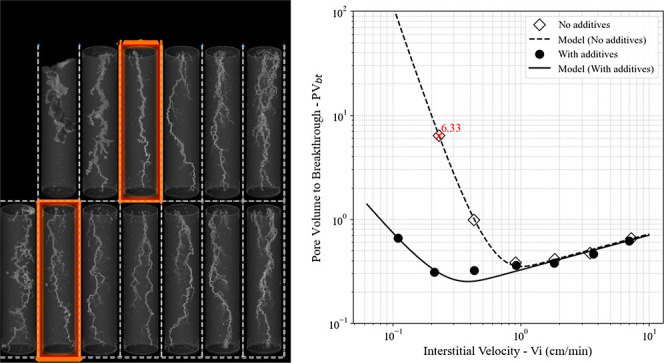

## 1 Introduction

The exploitation of hydrocarbon reservoirs is often challenged by formation damage near the wellbore, commonly referred to as the skin effect, which can significantly reduce well productivity even in high-porosity and high-permeability formations.^1–4^ Chemical stimulation, particularly acid stimulation, is widely applied to mitigate this damage by removing blockages and creating conductive flow channels, thereby enhancing permeability and hydrocarbon flow toward the wellbore.^5^

In carbonate reservoirs, hydrochloric acid (HCl) is commonly used due to its strong dissolution capacity, cost-effectiveness, and formation of water–soluble reaction products.^6–9^ However, its high reactivity can result in severe corrosion of wellbore equipment and excessively rapid acid–rock reactions, limiting acid penetration and increasing acid consumption under certain reservoir and operational conditions.^10,11^

In this scenario, analyzing the interaction between the reactive fluid and the rock is crucial for understanding the efficiency of the stimulation process. This interaction can be investigated in the laboratory by constructing pore volume to breakthrough (PV_bt_) curves, which correspond to the number of pore volumes of acid required to propagate through the full length of the rock sample, achieving breakthrough. The optimal point of the PV_bt_ curve, corresponding to the minimum acid volume needed to achieve breakthrough, can be determined by conducting core flooding experiments at different injection rates or interstitial velocities. Typically, core plugs with lengths ranging from 3 to 10 in. Are used in these studies.^12–14^

Beyond the acid–rock interaction, the overall performance of acidizing treatments can be significantly influenced by the composition of the injected fluid, particularly by the presence of specific additives. Two main groups of additives are especially relevant in reactive fluid formulations: corrosion inhibitors and emulsion preventers. From a production standpoint, it is also important to recognize that acidizing can lead to significant financial losses due to the corrosion of metallic infrastructure in oil wells. Such infrastructure, mainly composed of N80 carbon steel, is widely used because of its mechanical stability and economic viability.^15^ The corrosion of these materials during acidizing processes can significantly increase production costs. To mitigate this issue, corrosion inhibitors are typically added to the acid solution before injection.^16^

Corrosion inhibitors protect carbon steel surfaces and are generally composed of alcohols and amines.^7,17^ Quaternary amines, due to their positive charge, can adsorb onto the negatively charged steel surfaces, forming a protective layer that reduces corrosion in acidic environments. Alcohols with triple bonds, such as acetylenic alcohols or octynol, can also form protective films through chemical or physical interactions, creating a barrier that isolates the injected acid from the metallic surface.^18^ Emulsion preventers, on the other hand, modify the physicochemical properties at the interface between the acid and the formation fluids, thereby preventing the formation of undesirable emulsions. These additives ensure that the acid flows freely through the formation, improving its efficiency in removing mineral deposits and stimulating oil production.^19^

In addition, additives such as polymers, emulsifiers, foaming agents, and viscoelastic surfactants are frequently used to promote better acid diversion and deeper penetration, thereby optimizing the overall stimulation efficiency.^20^ The controlled dissolution promoted by certain additives is essential for optimizing wormhole formation. These compounds help regulate the reaction rate between the injected chemicals and the rock, ensuring that the development of channels occurs in a controlled manner.^21,22^

Given this context, this study aims to investigate possible changes in acid–rock interaction caused by the presence of additives whose primary functions are to inhibit corrosion and prevent emulsion formation. Because carbonate acidizing is sensitive to flow conditions, a series of core flooding experiments was conducted at different injection rates to evaluate how acid consumption varies as a function of interstitial velocity, as expressed by the PV_bt_ curve. This approach allows the identification of representative trends and optimal conditions rather than relying on isolated tests. Additionally, the influence of these additives on wormhole formation was assessed using X-ray microcomputed tomography (μCT).

The present work does not aim to introduce a new acidizing mechanism, but rather to quantitatively evaluate how a commercial corrosion inhibitor and emulsion preventer system, originally designed for corrosion and emulsion control, affects PV_bt_ behavior, optimal interstitial velocity, and the parameters of the Buijse–Glasbergen model during matrix acidizing of a highly calcitic carbonate rock. To isolate the effect of chemical additives under controlled flow conditions, pore structure is not treated as an independent variable, and no detailed pore structure analysis using advanced techniques such as mercury intrusion porosimetry or scanning electron microscopy is pursued.

## 2 Materials and Methods

### 2.1 Materials

The following reagents were used in the experiments: hydrochloric acid (HCl, 37 wt %, Synth, Brazil) and two commercial additives, namely a corrosion inhibitor and an emulsion preventer. Because of confidentiality agreements with the supplier, the detailed formulations of these additives cannot be reported. For the purposes of this study, it is relevant to note that the corrosion inhibitor consists mainly of surfactants, quaternary ammonium salts, alcohols, and aldehydes, whereas the emulsion preventer contains only nonionic surfactants and ethanol. These general compositional features are provided to help contextualize the physicochemical behavior discussed throughout this work, without compromising proprietary information. Commercial Indiana Limestone samples were obtained from Kocurek Industries, Inc. (Texas, USA). All aqueous solutions were prepared using distilled water.

#### 2.1.1 Preparation of Stimulation Fluid

For the preparation of thvde additive-free stimulation fluid, concentrated hydrochloric acid (37 wt %) was diluted to 15 wt % using distilled water. For the additive-containing formulation, the corrosion inhibitor (0.5 vol %) and the emulsion preventer (0.2 vol %) were first added to distilled water. Then, concentrated HCl (37 wt %) was added and diluted to reach a final concentration of 15 wt %.

#### 2.1.2 Dynamic Viscosity Determination

Viscosity analyses were performed to investigate the effect of additive concentration on the viscosity of the 15 wt % HCl solution. This property directly influences fluid flow behavior and the transport of H^+^ ions toward the rock surface.^23,24^ A Cannon–Fenske capillary viscometer was used inside a thermostatic bath maintained at 25 °C (ambient conditions). The viscosity of the solution was calculated using eq 1

1 $$\mu_{S}=\mu_{w}\frac{\rho_{s}t_{s}}{\rho_{w}t_{w}}$$

where μ_s_, ρ_s_, and *t*_s_ are the viscosity, density, and flow time of the solution, respectively; μ_w_, ρ_w_, and *t*_w_ are the corresponding parameters for distilled water.

#### 2.1.3 Surface Tension

Surface tension measurements were conducted using a Krüss K20 tensiometer (Hamburg, Germany) employing the Du Noüy ring method at room temperature (25 °C). This analysis aimed to evaluate the influence of additives on the surface tension of the reactive fluid, a property directly associated with interfacial phenomena. Changes in surface tension can promote or inhibit emulsion formation, which is generally considered undesirable in acid stimulation operations. Measurements were carried out for both additive-free and additive-containing acid solutions, and all tests were performed in triplicate to ensure reproducibility.

#### 2.1.4 Foam Formation and Stability Tests

Foam formation and stability were evaluated through bottle tests performed at ambient conditions, following the methodology proposed by.^25^ The acid solution containing additives was first transferred to a beaker, where CO_2_ was injected for 1 min at a flow rate of 10 L/min. This procedure was adopted to introduce a gaseous phase corresponding to that generated during carbonate dissolution. Gas injection was performed using a flexible tube moved in circular motions to promote effective dispersion of CO_2_ in the liquid phase.

For each test, 4 mL of the solution were transferred to 10 mL graduated cylinders, which were then sealed with plastic film. The samples were vigorously shaken for 30 s by the same operator to ensure reproducibility. Immediately after agitation, foam height was monitored at 1 min intervals to assess foam formation and stability over time. The experiments were terminated when the foam volume decreased to half of its initial value, corresponding to the foam half-life, and all experiments were conducted in triplicate.

### 2.2 X-ray Diffraction (XRD) and X-ray Fluorescence (XRF)

The X-ray diffraction (XRD) pattern was obtained using a Bruker D2 Phaser benchtop diffractometer equipped with a Lynxeye detector (Germany). The samples were analyzed under Bragg–Brentano geometry (θ–2θ) in the angular range of 10°–80°, with a step size of 0.02° and a scan rate of 3°/min. The radiation source was Cu Kα (λ = 1.54 Å). All analyses were performed at ambient conditions (25 °C). The XRD data were interpreted based on the ICSD (Inorganic Crystal Structure Database) and refined using the Rietveld method.^26^

The X-ray fluorescence (XRF) analysis was carried out using a Shimadzu EDX-720 energy-dispersive spectrometer. This technique was employed to determine the elemental composition of the Indiana Limestone samples, providing complementary information on their chemical purity and confirming the predominance of CaCO_3_ in the rock matrix.

### 2.3 Core Flooding Experiments

The core flooding experiments were performed using Indiana Limestone cores with a diameter of 1.5 in and a length of 6 in. Two sets of experiments were conducted: (1) using 15 wt % HCl solution, and (2) using the same acid concentration with the addition of a corrosion inhibitor (0.5 vol %) and an emulsion preventer (0.2 vol %). All experiments were carried out at 25 °C. Different injection rates were applied: 0.25, 0.5, 1, 2, 4, 8, and 16 mL/min. However, it was only possible to test all these rates with the additive-containing solution; the additive-free acid caused rock collapse at 0.5 mL/min.

The reactive flow experiments were conducted using a core flooding system made of Hastelloy C-276 alloy, a material highly resistant to corrosion. A schematic of the setup is shown in Figure 1. To maintain system stability and a constant flow rate between 0.25 and 16 mL/min, a Vindum Engineering dual-piston pump, model VP-12K, was employed, providing continuous and highly precise flow control. Each test consisted of four stages: (I) Core saturation with 4 wt % KCl brine. (II) Brine injection at 2 mL/min until steady-state flow conditions were achieved. This allowed determination of the absolute permeability of the core using Darcy’s law for linear flow (eq 2).

2 $$k=\frac{q\cdot \mu \cdot L}{A\cdot \Delta p}$$

where *q* is the injection flow rate, *L* and *A* are the length and cross-sectional area of the core, respectively, μ is the brine viscosity at the experimental temperature, and Δ*P* is the pressure difference between the inlet and outlet of the core sample.

(III) The acid was then injected at a predetermined rate until it fully penetrated the rock. During the experiment, changes in differential pressure and effluent pH were monitored to track wormhole development and identify the breakthrough point. For Indiana Limestone cores acidized with HCl, breakthrough was identified by a sharp decrease in differential pressure to residual levels and a drop in effluent pH toward the injected acid value. The injected acid volume was corrected for the dead volume in the lines to determine the effective volume that passed through the rock core. (IV) After acidizing, brine was reinjected at 10 mL/min to remove any residual acid and stop further reactions with the carbonate matrix, preventing distortion in subsequent analyses such as microcomputed tomography. All experiments were carried out under the following conditions: a temperature of 25 °C, axial pressure of 500 psi, confining pressure of 2000 psi, and backpressure of 1200 psi, ensuring system stability throughout the tests.

**Figure 1:**
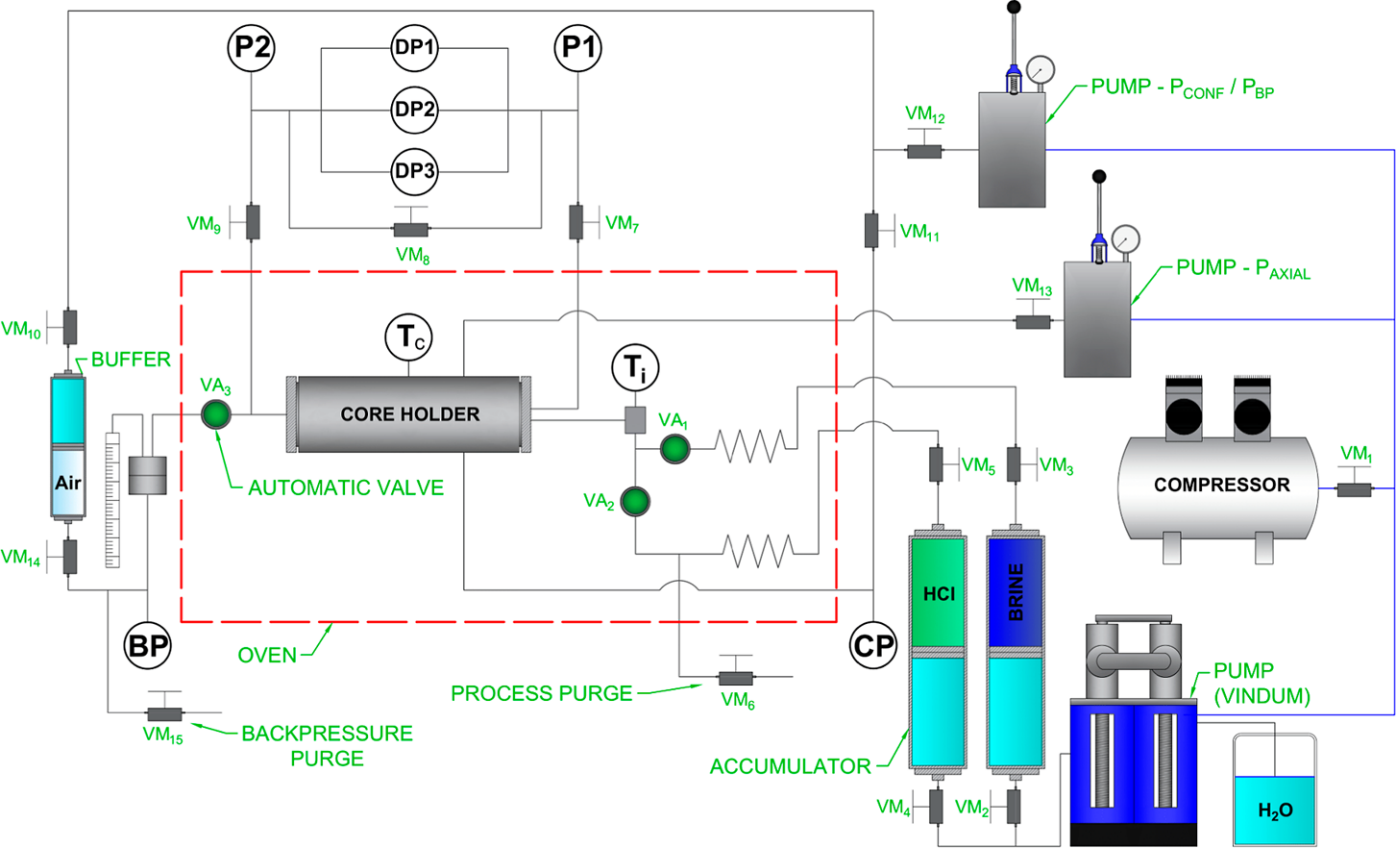
Process flowchart of the core flooding experiment.

The efficiency of acid stimulation was evaluated based on the number of pore volumes of acid required to reach breakthrough (eq 3) as a function of flow rate or interstitial velocity (*V*_*i*_), as expressed by eq 4.

3 $${PV}_{bt}=\frac{V_{inj}}{PV_{i}}$$

4 $$V_{i}=\frac{q}{\frac{1}{4}\pi d_{core}^{2}\cdot \phi }$$

The number of pore volumes to breakthrough (PV_bt_) was calculated by dividing the injected acid volume (*V*_inj_) by the initial pore volume of the sample (PV_i_). Regarding the *V*_*i*_, *q* is the volumetric acid injection rate, *d*_core_ is the core diameter, and ϕ is the core porosity.^27^ These parameters are mainly influenced by the reaction kinetics between the stimulation fluid and the rock, as well as by the injection rate.

### 2.4 μCT Analysis

The sample images were produced with a Nikon XT H 225 ST tomography equipped with a tungsten X-ray source. The scanning time for all projections was approximately 25 min with an individual projection time of 500 ms. The acquisition parameters were: current of 80 μA, voltage of 150 kV, additional 0.25 mm aluminum filter. The pixel resolution of all images was 60 μm.

After the samples scanning, the projections were reconstructed into three-dimensional data sets using the CT Pro 3D software. The 3D image volumes were analyzed with VG Studio Max 3.4.4, where each core’s orientation was adjusted to ensure maximum orthogonality relative to the lower plug face. A cylindrical sub volume was extracted, maximizing its size while compensating for shape irregularities in each sample.

To minimize image noise and enhance the accuracy of segmentation and digital petrophysical analysis, a median filter was applied.

Segmentation was then performed to define the pore network and the solid matrix. In the μCT images, light regions correspond to pore space and wormholes, whereas dark regions represent the carbonate matrix. This nondestructive imaging technique provided essential information on the spatial distribution and morphology of the dissolution channels formed under different injection conditions.

## 3 Results and Discussion

### 3.1 Viscosity, Surface Tension and Foam Stability of the Stimulation Fluid

The results showed that the viscosity of the 15 wt % HCl solution was approximately 1.216 cP, whereas the HCl solution containing additives exhibited a viscosity of 1.261 cP. Although slightly higher, the viscosity of the additive-containing formulation did not represent a significant increase compared to the plain acid, suggesting that the additives did not substantially alter the rheological behavior of the solution. These values are consistent with those reported in the literature for additive-free HCl solutions at the same concentration,^28^ confirming the reliability of the experimental data.

Regarding surface tension, the additive-free formulation presented a value of 66.01 mN/m, while the additive-containing formulation showed 31.34 mN/m. The reduction in surface tension indicates that the additives act as surfactants, as expected from their chemical composition. These molecules exhibit interfacial activity and can influence emulsion formation when the acid solution encounters oil, either promoting or suppressing it, as reported by Ganeeva et al.^29^ and Rabie & Nasr-El-Din.^30^ Emulsion formation is generally undesirable, as it decreases the mobility of the fluid through the porous medium.

In addition, the reduction in surface tension is known to facilitate foam generation and stabilization in systems where gas is present, as surfactants reduce the interfacial energy required for bubble nucleation and delay bubble coalescence.^31^ Consistent with this behavior, the acid solution with additives exhibited foam formation in the presence of CO_2_, with initial foam heights of 4.2 and 4.3 cm and foam half-life values of 47 and 48 min in replicate tests. These results indicate that the system can sustain a dispersed gas phase over time scales relevant to acidizing. Thus, transient foam formation may act as a local transport barrier, restricting H^+^ diffusion toward the rock surface and moderating the acid–rock reaction rate, which may help explain the differences observed in PV_bt_ behavior between formulations with and without additives.

### 3.2 Mineralogical Analysis of Carbonate Rocks

Figure 2 shows the X-ray diffraction (XRD) pattern obtained for the carbonate rock. The diffraction peaks were clearly identified and correspond to the characteristic fingerprint of CaCO_3_. The main diffraction peaks appeared at 2θ angles of 23.06°, 29.46°, 36.00°, 39.44°, 43.20°, 47.50°, 48.58°, and 57.46°, associated with the crystallographic planes (012), (104), (110), (113), (202), (018), (116), and (122) of calcite, respectively. These results agree with standard XRD data and previous studies on pure calcium carbonate.^32^ The Rietveld refinement was performed using the MAUD software.^33^ The analysis indicated a hexagonal crystal structure with *a* space group *R*3̅*C* and unit cell parameters *a* = 4.98 Å, *c* = 17.04 Å, γ = 120°, consistent with the literature.^34^ These parameters confirm that the sample is composed exclusively of calcite (CaCO_3_), with no evidence of other mineral phases.

**Figure 2:**
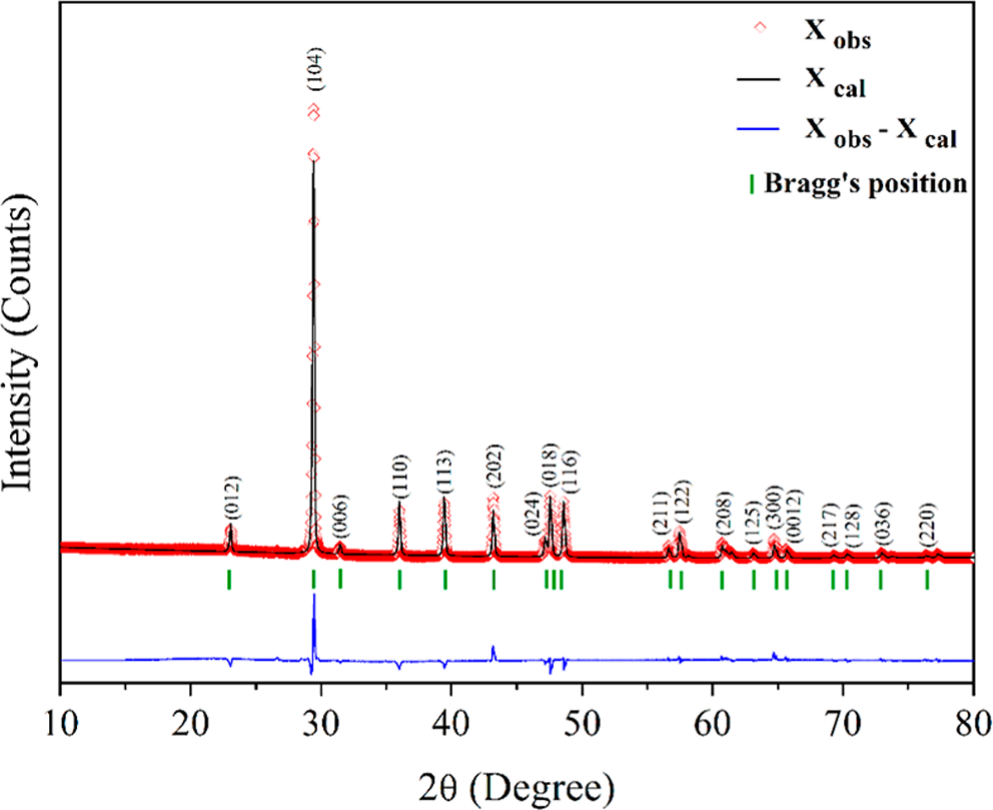
–Rietveld refinement profile of the XRD data for CaCO_3_. The red dots represent the experimental data, the black solid line indicates the calculated pattern, and the blue curve at the bottom represents the difference between the observed and calculated.

The chemical composition of the Indiana Limestone samples was determined by X-ray fluorescence (XRF). The results, expressed as oxide weight percentages, are presented in Table 1.

**Table 1:** Chemical Composition (wt %) of Indiana Limestone

analyte	result (%)	standard deviation
CaO	98.578	0.225
K_2_O	0.397	0.034
Al_2_O_3_	0.305	0.041
SiO_2_	0.280	0.019
SrO	0.268	0.018
SO_3_	0.123	0.012
CuO	0.049	0.010

A high CaO content (98.578 wt %), in agreement with the XRD results, confirms that the rock consists almost entirely of calcite with negligible contributions from impurity minerals (1.422 wt %), so dissolution follows the classical reaction between calcite and HCl (eq 5).

5 $$CaC{O_{3}}_{\left(s\right)}+2HCl_{\left(aq\right)}\to CaC{l_{2}}_{\left(aq\right)}+{CO_{2}}_{\left(g\right)}^{↑}+H_{2}O_{\left(l\right)}$$

### 3.3 Core Flooding Analysis

Table 2 summarizes the petrophysical properties of the rock samples, including porosity and absolute permeability, along with the different injection rates and pore volume to breakthrough (PV_bt_) values obtained for each acid formulation.

**Table 2:** Petrophysical and Experimental Results for the Core Flooding Tests

sample	solution	porosity (%)	permeability (mD)	*V*_*i*_ (cm/min)	*q* (mL/min)	PV_bt_
01	HCl 15%	20.10	79.73	0.23	0.5	> 6.33
02	HCl 15%	20.24	69.35	0.43	1	0.98
03	HCl 15%	19.29	96.93	0.91	2	0.38
04	HCl 15%	19.10	60.32	1.84	4	0.41
05	HCl 15%	20.21	73.87	3.47	8	0.47
06	HCl 15%	19.28	68.75	7.28	16	0.67
07	HCl 15% + additives	20.17	45.32	0.11	0.25	0.66
08	HCl 15% + additives	20.58	75.90	0.21	0.5	0.31
09	HCl 15% + additives	20.37	79.90	0.43	1	0.32
10	HCl 15% + additives	19.10	51.34	0.92	2	0.36
11	HCl 15% + additives	19.26	71.87	1.82	4	0.38
12	HCl 15% + additives	19.15	58.66	3.66	8	0.46
13	HCl 15% + additives	20.01	36.26	7.01	16	0.62

In terms of basic petrophysical properties, the porosity values obtained for the 13 samples showed limited variation, ranging from 19.10% to 20.58%, with an average of 19.76 ± 0.56%. This narrow range suggests a relatively consistent pore volume among the analyzed plugs. To account for differences between individual cores, all acid volumes to breakthrough and injection rates were normalized using the individual pore volume of each core sample, as defined in Equations 3 and ([Disp-formula eq4]), respectively.

Permeability values ranged from 36.26 mD to 96.93 mD, with an average of 66.78 ± 16.22 mD. Despite minor fluctuations, all samples exhibited values of the same order of magnitude.

The remaining results, summarized in Table 2, were plotted in Figure 3 as PV_bt_ versus interstitial velocity (*V*_*i*_) curves, allowing visualization of the trend between these variables.

**Figure 3:**
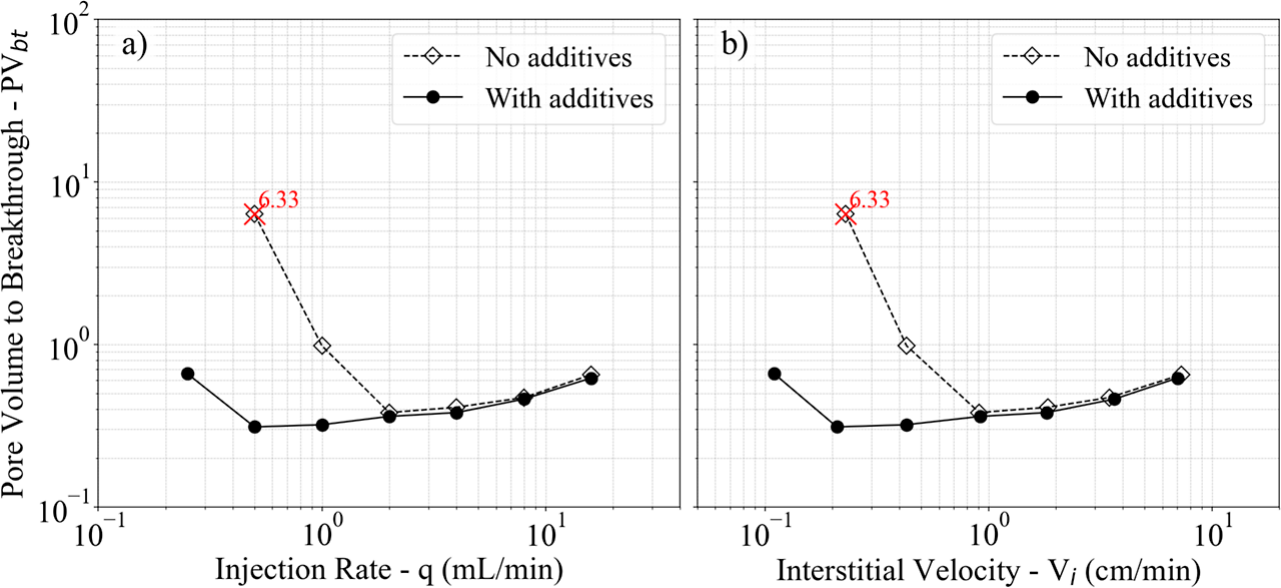
Pore volume to breakthrough (PV_bt_) curves as a function of (a) injection rate and (b) interstitial velocity.

#### 3.3.1 Pore Volume to Breakthrough (PV_bt_) Curves

Based on the reactive flow tests performed for each stimulation fluid at different injection rates (as shown in Table 2), it was possible to plot the PV_bt_ vs *V*_*i*_ curves, as illustrated in Figure 3. The objective was to determine the optimal injection rate (*V*_*i*‑opt_) at which the minimum acid consumption required to penetrate the rock (PV_bt‑opt_) is observed, resulting in higher process efficiency. This procedure was carried out for both the conventional 15 wt % HCl solution (without additives) and for the same solution containing the emulsion preventer and corrosion inhibitor.

As shown in Figure 3, plotted on a logarithmic scale on both axes (log–log), the stimulation fluids exhibited similar behavior under experimental conditions from 2 mL/min onward (*V*_*i*_ ≈ 0.91 cm/min). In this range, the PV_bt_ curve tends to stabilize, indicating comparable performance between the formulations with and without additives. However, at lower flow rates, an increase in acid consumption was observed in the absence of additives. This difference is so significant that, while the additive-containing system presented an experimentally observed PV_bt‑opt_ at 0.5 mL/min, the additive-free fluid, represented in Figure 3 by the red “×” symbol, consumed about 6.33 pore volumes of acid and failed to reach breakthrough, requiring the experiment to be paused to preserve equipment integrity.

As observed in Figure 3, the presence of additives led to a substantial reduction in the optimal interstitial velocity (*V*_*i*‑opt_), from 0.91 cm/min (without additives) to 0.21 cm/min (with additives), corresponding to an approximately 77% decrease. It is important to note that this change was achieved without a significant increase in fluid viscosity.

This reduction can be attributed to the influence of additives on the system’s reactivity, which tends to reduce the acid reaction rate near the sample inlet. Consequently, the acid can travel longer distances before being consumed, favoring the formation of deeper wormholes even under low interstitial velocity conditions.

According to the model proposed by Buijse and Glasbergen,^27^ the pore volume to breakthrough (previously described in eq 3) can be further adapted to eq 6, which expresses the relationship in terms of the interstitial acid velocity and the wormhole growth behavior.

6 $$PV_{bt}=\frac{V_{i}}{V_{wh}}$$

where *V*_*i*_ is the interstitial velocity (eq 4), and *V*_wh_ represents the wormhole front velocity expressed in eq 7

7 $$V_{wh}=W_{eff}\cdot V_{i}^{2/3}\cdot B\left(V_{i}\right)$$

with *V*_*i*_ and *V*_wh_ properly defined, eq 6 can be reformulated as eq 8

8 $$PV_{bt}=\frac{Vi}{V_{wh}}=\frac{V_{i}^{1/3}}{W_{eff}\cdot B\left(V_{i}\right)}$$

where B­(*V*_*i*_) is defined by eq 9

9 $$B\left(V_{i}\right)={\left(1-e^{\left(-W_{B}\cdot V_{i}^{2}\right)}\right)}^{2}$$

Equations 8 and 9 can be combined to yield eq 10, which expresses PV_bt_ as a function of *V*_*i*_, *W*_eff_, and *W*_B_

10 $$PV_{bt}=\frac{V_{i}^{1/3}}{W_{eff}{\cdot \left(1-e^{\left(-W_{B}\cdot V_{i}^{2}\right)}\right)}^{n}}$$

The parameters *W*_eff_ (wormhole opening efficiency factor) and *W*_B_ (wormhole growth factor) are constants that can be obtained by fitting eq 10 to the experimental data. According to the model, the exponent *n* is equal to 2. Alternatively, both parameters can be estimated from the optimal interstitial velocity (related to the ideal injection rate) and PV_bt_ values using eqs 11 and 12, although this approach provides less accurate results.

11 $$W_{eff}=\frac{V_{i-opt}^{1/3}}{{PV}_{bt-opt}}$$

12 $$W_{B}=\frac{4}{V_{i-opt}^{2}}$$

Once these constants are determined for each experiment, it becomes possible to generate the PV_bt_ curve and estimate its value at different injection rates. From the fitted curves, the optimal PV_bt_ value can be identified.^27,35,36^ In order to assess the influence of each factor on PV_bt_ behavior, arbitrary values were assigned to *n*, *W*_eff_, and *W*_B_ for plotting Figure 4. The reference condition corresponds to *W*_eff_ = 2, *W*_B_ = 10^0^, and *n* = 2, which together define the black curve. Subsequently, each parameter was varied independently to analyze its individual effect. This analysis provides the basis for the discussion of the results obtained.

**Figure 4:**
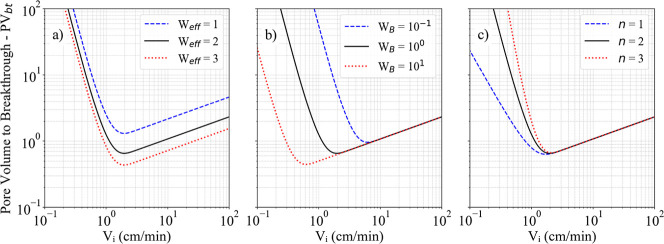
Dependence of the PV_bt_ curve on variations in Buijse–Glasbergen model parameters – (a) *W*_eff_, (b) *W*_B_, (c) *n*.

Changes in *W*_eff_ (Figure 4a) indicate that increasing this factor uniformly reduces PV_bt_ along the entire curve, improving overall dissolution performance while preserving the position of *V*_*i*‑opt_. Although both *W*_eff_ and *W*_B_ depend on *V*_*i*‑opt_, only *W*_eff_ is directly related to the PV_bt‑opt_. Consequently, varying *W*_eff_ with constant *W*_B_ only results in a vertical shift of the curve without altering its shape or the corresponding *V*_*i*_.

The variation of *W*_B_ (Figure 4b) shows that increasing this parameter shifts the optimal condition toward lower interstitial velocities and lower PV_bt_ values, without significant changes in the region with high interstitial velocity.

The variation of *n* (Figure 4c) shows that increasing this exponent narrows the optimal region and increases acid consumption at low injection rates, where face dissolution dominates. Higher *n* values therefore accentuate the contrast between inefficient and efficient regimes, making the system more sensitive to small variations in *V*_*i*_ below the optimum condition.

The experimental data fitted to the Buijse–Glasbergen model exhibited good agreement for both conditions, with and without additives, as illustrated in Figure 5. Table 3 summarizes the results obtained from the fitting procedure.

**Figure 5:**
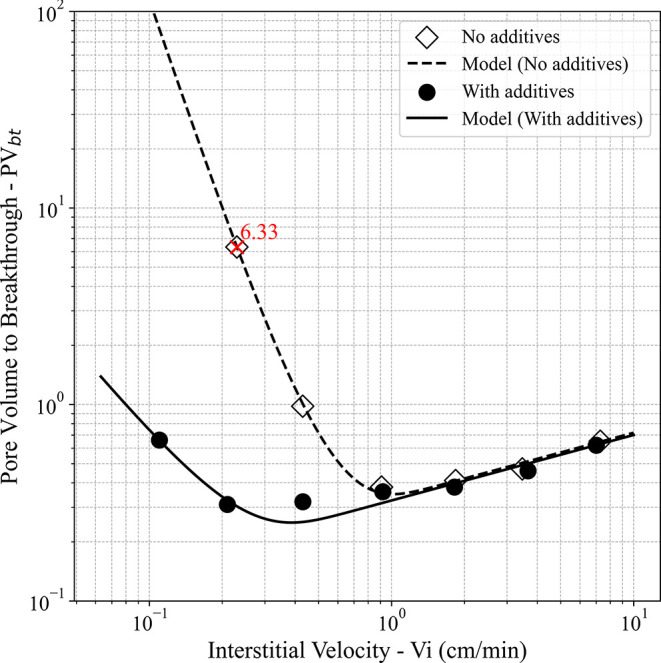
Pore volume to breakthrough (PV_bt_) curves as a function of interstitial velocity for systems with and without additives, compared to the Buijse–Glasbergen model predictions.

**Table 3:** Fitting Parameters (*W*_eff_, *W*_B_, *n* and Correlation Coefficients), *V*_*i*‑opt_, and PV_bt‑opt_ Obtained From the Buijse–Glasbergen Model for Systems With and Without Additives

solution	*W*_eff_ (cm/min)^1/3^	*W*_B_ (cm/min)^−2^	*n*	*R* ^2^	*V*_*i*‑opt_ (cm/min)	PV_bt‑opt_
HCl 15%	3.00	3.74	2.00	0.99	1.02	0.35
HCl 15% + additives	3.08	18.54	0.90	0.93	0.46	0.26

According to Table 3, both fluids presented significantly close values of *W*_eff_ (3.00 for 15% HCl and 3.08 for 15% HCl + additives), indicating a slightly higher overall efficiency for the additive-containing system, consistent with the overlapping curves in the high interstitial velocity region, as observed in Figure 5.

The main change in curve shape and optimum position, however, was caused by *W*_B_ (3.74 for 15% HCl and 18.54 for 15% HCl + additives), whose value increased by almost 5-fold. As also illustrated in Figure 5, this increase shifts the optimum point diagonally toward the lower left, indicating a simultaneous decrease in PV_bt–opt_ and *V*_*i*–opt_, while the high interstitial velocity region remains practically unchanged.

Regarding exponent *n*, in the additive-containing formulation, the original Buijse–Glasbergen model^27^ with an exponent equal to 2 (eq 10) failed to adequately reproduce the experimental data. This empirical exponent directly controls the slope of the PV_bt_ curve at low flow rates: higher exponent values (e.g., 2) produce steeper curves (as previously shown in Figure 4), which might represent strong acids well but do not fit less reactive systems.

In the present study, the presence of the corrosion inhibitor and emulsion preventer reduced the acid–rock reaction rate, producing smoother experimental curves at low flow rates and compromising the fit of the classical model. To overcome this limitation, the fitting process was refined by simultaneously adjusting the exponent *n* of B­(*V*_*i*_), *W*_eff_, and *W*_B_ to obtain a better fit between the model and the experimental data. This adjustment reduced the exponent *n* from 2 to 0.9 and improved the coefficient of determination (*R* ^2^) from 0.85 to 0.93 by softening the curve slope, which more accurately represented the behavior of HCl containing additives.

This result demonstrates that adjusting the exponent broadens the model’s applicability, allowing an accurate description of systems with lower reactivity and reinforcing the importance of empirical flexibility when applying the Buijse–Glasbergen model to modified acid formulations.

The change in wormhole-formation behavior in the presence of additives has also been reported in previous studies,^8,37,38^ where the authors investigated the influence of nonionic surfactants on the reaction between reactive fluid formulations and carbonate rocks. The formation of CO_2_ gas bubbles, products of the acid–rock reaction occurring in the presence of surfactants, leads to foam generation,^39,40^ which acts as a gas layer that creates a barrier between the rock and the reactive fluid, thereby limiting the contact of H^+^ ions with the rock surface. This barrier may form during the reaction, promoting dissolution control even at low flow rates. In contrast, in the absence of additives, the longer contact time between acid and rock results in excessive surface reaction, as observed in additive-free experiments.

In addition, several studies have demonstrated that adsorption of organic additives can also induce a retardation effect during carbonate acidizing. Compounds present in corrosion inhibitors and emulsion preventers, such as surfactants, can adsorb onto the carbonate surface, forming a film that might act as a diffusive barrier. The adsorbed layer can limit the access of H^+^ ions to the reactive sites of the carbonate rock.^41–44^

This mechanism of retard complements the physical barrier created by CO_2_ foam, jointly contributing to acid-reaction control even at low flow velocities. Therefore, additives positively influence the development of flow channels, particularly at suboptimal flow rates.

#### 3.3.2 Analyses of the Core Inlet Faces after Acidizing

Figure 6a shows the inlet faces of the cores after the acidizing stage. It is evident that, at low injection rates without additives, excessive dissolution occurred on the inlet face. However, this phenomenon was not observed at flow rates above 2 mL/min. The overly high dissolution of the rock sample surfaces resulted from the high reaction rate between HCl and the carbonate matrix (eq 5), combined with the longer contact time between the reactive fluid and the rock.

**Figure 6:**
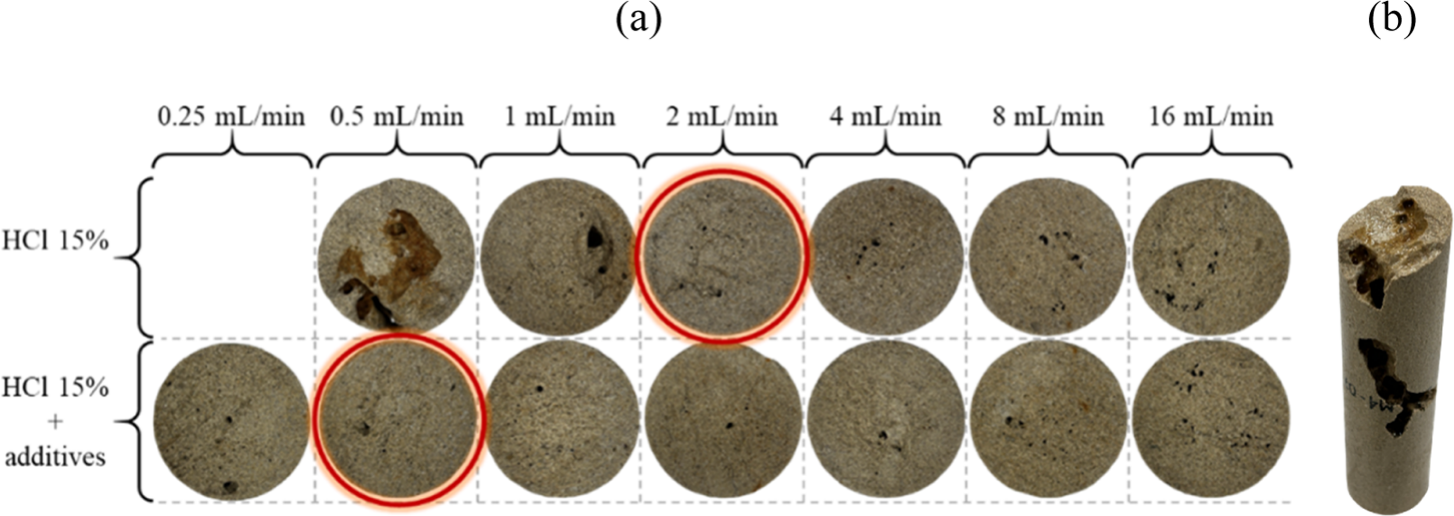
(a) Images of the core inlet faces after acidizing. (b) Sample acidized with 15 wt % HCl without additives at a flow rate of 0.5 mL/min.

Compared to higher injection rates, the surface damage was reduced due to the shorter contact time, which facilitated acid flow through the rock and contributed to wormhole formation. The cores highlighted with red circles correspond to the experimental conditions that exhibited the lowest PV_bt_ values. Their surfaces remain preserved, with dissolution limited to the wormhole formation. This morphology indicates more efficient wormhole propagation immediately after acid entry, favoring the formation of a single and dominant flow channel, as later confirmed by microtomography results.

In Figure 6b, the sample corresponding to the experiment performed at 0.5 mL/min without additives is shown. The image reveals enlarged and inefficient dissolution channels, as well as areas severely affected by acid dissolution, resulting in structural loss at the inlet face and along the core sample. This behavior is characteristic of inefficient acidizing, caused by the prolonged contact time at low flow rates and by the high dissolution kinetics of the carbonate in HCl under the studied conditions.

These effects were also confirmed by the μCT images presented in Figure 7, which reveal a thick and poorly developed wormhole that failed to reach breakthrough. These results are consistent with the PV_bt_ curve shown in Figure 5, which required interruption of the test, demonstrating the significant impact of additive absence on the rock dissolution.

**Figure 7:**
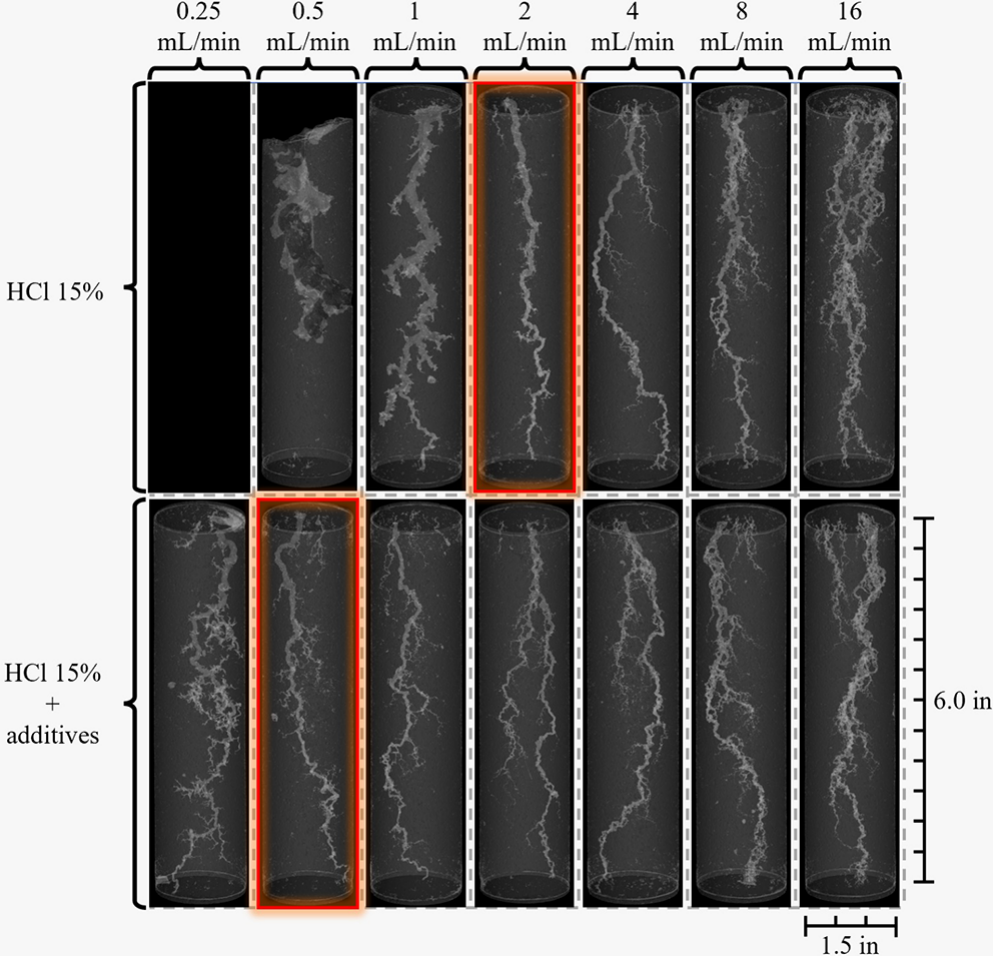
X-ray microcomputed tomography images of the cores acidized with and without additives.

The acid solutions containing additives prevented excessive dissolution of the inlet faces at any of the injection rates evaluated. As previously discussed, this behavior can be attributed to the reduced diffusion of H^+^ ions caused by the presence of additives, which limits the direct contact of the acid with the rock surface and slows its dissolution. Consequently, the H^+^ ions can travel further through the porous medium before being consumed at the surface, facilitating acid penetration into the sample.^8^

#### 3.3.3 Computed Microtomography Analysis

Figure 7 presents the μCT images used to evaluate the wormhole patterns formed in Indiana Limestone samples after acidizing with different solutions, 15 wt % HCl without additives and 15 wt % HCl with additives, under several injection rates. The samples with the highest efficiency (PV_bt‑opt_) in each acid system are highlighted in red. This analysis allows for a clearer comparison between the performances of the two systems.

The samples highlighted in red indicate that, in the absence of additives, the optimal wormhole formation occurred only at an injection rate of 2 mL/min, whereas the formulation containing additives significantly expanded the range of optimal flow rates, displaying dominant channels starting at just 0.5 mL/min. This behavior demonstrates the effect of the additives to control the acid–rock reactivity and to promote the development of dominant dissolution channels under various flow conditions. In the velocity region, for both acid formulations, a significant increase in the number of secondary channels is observed at flow rates above 4 mL/min, becoming even more pronounced at the highest rate tested in this study (16 mL/min).

The results presented here are consistent with those reported by Rodrigues et al.,^8,38^ who emphasized the effectiveness of chemical additives in optimizing acid stimulation processes in carbonate rocks. These additives can directly influence dissolution efficiency through mechanisms such as reaction-rate control and the formation of more uniform dissolution structures. Rodrigues et al. and Arruda et al.,^8^ observed, through μCT imaging, that solutions containing highly ethoxylated nonionic surfactants, which cause greater reaction retardation,^45^ generated dominant wormhole patterns with the lowest PV_bt_ and *V*_*i*_, compared with plain HCl injections, which produced conical channels at lower interstitial velocity.

These findings reinforce the potential of such formulations for applications in carbonate acidizing treatments, as despite being primarily intended for corrosion inhibition and emulsion prevention, these additives exert a secondary influence on the reaction kinetics and, more importantly, on the PV_bt_ curves and resulting wormhole patterns.

## 4 Conclusion

The core flooding experiments clearly demonstrated the direct impact of additives on the efficiency of the acidizing process. The pore volume to breakthrough (PV_bt_) curves indicated a significant reduction in PV_bt_ values when additives were present, particularly at low injection rates, with a decrease in PV_bt‑opt_ from 0.35 to 0.26 and a reduction in the optimal interstitial velocity (*V*_*i*‑opt_) of approximately 77% (from 0.91 to 0.21 cm/min), without affecting the fluid viscosity (variation lower than 4%). Moreover, operating at lower flow rates reduces the risk of rock fracturing and decreases pump demand, which can be advantageous in field applications.

The μCT analyses revealed the consistent formation of dominant wormholes at most of the tested flow rates for the additive-containing system, whereas the samples treated with additive-free HCl exhibited less efficient dissolution patterns or even structural collapse under low-flow conditions, consuming more than 6 pore volumes of acid without reaching breakthrough at 0.5 mL/min. These findings demonstrate that, although traditionally used to inhibit corrosion and prevent emulsion formation, the additives employed here played an additional role in modifying the acid–rock reaction. This modification facilitated the formation of efficient flow channels within a highly reactive, calcite-rich matrix, thereby broadening the operational window for carbonate reservoir treatments.

Therefore, the acid formulation containing these commercial additives shows strong potential for practical application, enabling more efficient and predictable acidizing operations. The behavior observed for HCl with additives tends to enhance the outcome of matrix acidizing, particularly in low-injectivity scenarios, where the acid requires additional time to penetrate the formation and gradually improve injectivity. In such cases, the injection pressure cannot be substantially increased to avoid unwanted fracturing, and the system operates below the optimal interstitial velocity, which may lead to prolonged acid–rock contact and, consequently, face dissolution. This effect can be more pronounced in formations with higher temperatures. In such cases, the corrosion inhibitor and emulsion preventer act synergistically as retarders, reducing the dissolution rate and promoting the formation of deeper wormholes even at lower flow rates.

The results presented in this study apply primarily to highly calcitic matrices under laboratory temperature (25 °C). In carbonate reservoirs with different mineralogies, such as dolomite or mixed carbonate systems, or at high temperatures, changes in dissolution kinetics and acid–rock mass transfer may influence the degree of reaction retardation provided by the additives. Therefore, while the trends observed here provide useful mechanistic insights, their direct application to field-scale operations requires reservoir specific evaluation.

Although the detailed chemical composition of the additives is proprietary, the quantitative trends reported here demonstrate that the addition of chemical additives originally designed for other operational purposes can significantly alter PV_bt_ behavior. These results highlight the importance of evaluating any modification in acid composition through core flooding experiments prior to field application.

Future studies will evaluate the effect of different additive concentrations to improve the efficiency of acidizing and reduce treatment costs without compromising their primary functions of corrosion inhibition and emulsion prevention. However, this approach should be further evaluated considering the cost of the corrosion inhibitor and the emulsion preventer relative to other retarded acid systems, as well as their potential impact on formation damage. The individual contribution of each additive will also be investigated to better understand their specific role in the acid–rock interaction. In addition, the influence of permeability, mineralogy, and temperature on the performance of these additives in HCl-based systems will be further assessed.
